# Global Surveillance of Emerging Influenza Virus Genotypes by Mass Spectrometry

**DOI:** 10.1371/journal.pone.0000489

**Published:** 2007-05-30

**Authors:** Rangarajan Sampath, Kevin L. Russell, Christian Massire, Mark W. Eshoo, Vanessa Harpin, Lawrence B. Blyn, Rachael Melton, Cristina Ivy, Thuy Pennella, Feng Li, Harold Levene, Thomas A. Hall, Brian Libby, Nancy Fan, Demetrius J. Walcott, Raymond Ranken, Michael Pear, Amy Schink, Jose Gutierrez, Jared Drader, David Moore, David Metzgar, Lynda Addington, Richard Rothman, Charlotte A. Gaydos, Samuel Yang, Kirsten St. George, Meghan E. Fuschino, Amy B. Dean, David E. Stallknecht, Ginger Goekjian, Samuel Yingst, Marshall Monteville, Magdi D. Saad, Chris A. Whitehouse, Carson Baldwin, Karl H. Rudnick, Steven A. Hofstadler, Stanley M. Lemon, David J. Ecker

**Affiliations:** 1 Ibis Biosciences Inc., A Wholly Owned Subsidiary of Isis Pharmaceuticals, Carlsbad, California, United States of America; 2 Naval Respiratory Disease Laboratory, Naval Health Research Center, San Diego, California, United States of America; 3 Department of Emergency Medicine and Medicine, The Johns Hopkins Medical Institutions, Baltimore, Maryland, United States of America; 4 Wadsworth Center, New York State Department of Health, Albany, New York, United States of America; 5 College of Veterinary Medicine, The University of Georgia, Athens, Georgia, United States of America; 6 Naval Medical Research Unit #3, Cairo, Egypt; 7 United States Army Medical Research in Infectious Diseases, Ft. Detrick, Maryland, United States of America; 8 Science Applications International Corporation, San Diego, California, United States of America; 9 Institute for Human Infections and Immunity, University of Texas Medical Branch, Galveston, Texas, United States of America; US Naval Medical Research Center Detachment/Centers for Disease Control, United States of America

## Abstract

**Background:**

Effective influenza surveillance requires new methods capable of rapid and inexpensive genomic analysis of evolving viral species for pandemic preparedness, to understand the evolution of circulating viral species, and for vaccine strain selection. We have developed one such approach based on previously described broad-range reverse transcription PCR/electrospray ionization mass spectrometry (RT-PCR/ESI-MS) technology.

**Methods and Principal Findings:**

Analysis of base compositions of RT-PCR amplicons from influenza core gene segments (PB1, PB2, PA, M, NS, NP) are used to provide sub-species identification and infer influenza virus H and N subtypes. Using this approach, we detected and correctly identified 92 mammalian and avian influenza isolates, representing 30 different H and N types, including 29 avian H5N1 isolates. Further, direct analysis of 656 human clinical respiratory specimens collected over a seven-year period (1999–2006) showed correct identification of the viral species and subtypes with >97% sensitivity and specificity. Base composition derived clusters inferred from this analysis showed 100% concordance to previously established clades. Ongoing surveillance of samples from the recent influenza virus seasons (2005–2006) showed evidence for emergence and establishment of new genotypes of circulating H3N2 strains worldwide. Mixed viral quasispecies were found in approximately 1% of these recent samples providing a view into viral evolution.

**Conclusion/Significance:**

Thus, rapid RT-PCR/ESI-MS analysis can be used to simultaneously identify all species of influenza viruses with clade-level resolution, identify mixed viral populations and monitor global spread and emergence of novel viral genotypes. This high-throughput method promises to become an integral component of influenza surveillance.

## Introduction

Influenza viruses cause serious global economic and public health burdens. Annual influenza epidemics resulted in more than 30,000 deaths a year in the United States during 1990–1999[1], [2]. Periodic pandemics result in significantly higher death tolls. Emergence of new influenza A virus strains can be caused by “antigenic shift,” resulting from reassortment of gene segments, including H and/or N types[3], [4], “antigenic drift” resulting from the continuing accumulation of mutations in the H and N genes[5], or a pathogenic virus jumping species and acquiring the ability to infect and be transmitted among humans, as in the 1918 pandemic[6]. The recent outbreak of highly pathogenic H5N1 avian influenza virus (HPAI), which originated in Southeast Asia and has since spread globally, has resulted in 166 deaths (272 confirmed human cases) as of February 6, 2007 (http://www.who.int/en/). The global emergence of this virus has brought renewed urgency to the effort to track the spread and the evolution of influenza viruses.

Currently, rapid methods for influenza virus diagnosis rely on antigen-specific antibody probes[7], or real-time reverse transcription PCR (RT-PCR) analysis of the matrix (M) gene for identification of the viral species[8], [9] followed by H and N sub-type specific RT-PCR assays for determination of the viral sub-types[10], [11]. Since there are many H and N subtypes with significant intra- and inter-subtype sequence variations, these methods do not identify all H and N subtypes, nor are they likely to identify reassortants or newly emerging genetic variants. Further, none of the current surveillance methods provide information relevant to tracking antigenically novel strains that emerge each year or distinguish amongst multiple lineages of influenza viruses that can co-circulate and persist in a population[12]. Secondary genome sequence comparisons and phylogenetic analyses are necessary to fully understand the multiple lineages of viruses, recognize newly emergent influenza variants, and monitor global spread of these viruses[12], [13]. For instance, analysis of human influenza virus H3N2 sequences from 1999–2004 revealed that at least three major clades of influenza viruses were in circulation after the 2002–2003 influenza season[12]. The differences were due to multiple reassortment events though all shared a common H-gene lineage. Several similar whole-genome studies with avian influenza viruses have revealed the presence of multiple, region-specific sub-lineages of the HPAI H5N1 virus in Southeast Asia that are spreading to Europe and Africa[14]–[18].

We have developed a method based on broad-range RT-PCR followed by electrospray ionization mass spectrometry (RT-PCR/ESI-MS) for rapid and accurate detection of influenza virus, sub-species characterization, and early identification of genetic changes in circulating viruses. This method has previously been applied to detection of other pathogens in human clinical samples[19], [20], [21], [22], but it has unique capabilities and advantages for influenza surveillance. Here, we show how a high-throughput assay incorporating eight parallel RT-PCR reactions followed by ESI-MS analysis can be used to simultaneously survey for all species of influenza viruses, provide clade-level resolution, identify mixed viral populations in the same sample, detect reassortants, and facilitate monitoring of viral evolution, all integral components of broad influenza surveillance.

## Results

### Detection of influenza virus by RT-PCR/ESI-MS

**Figure 1:**
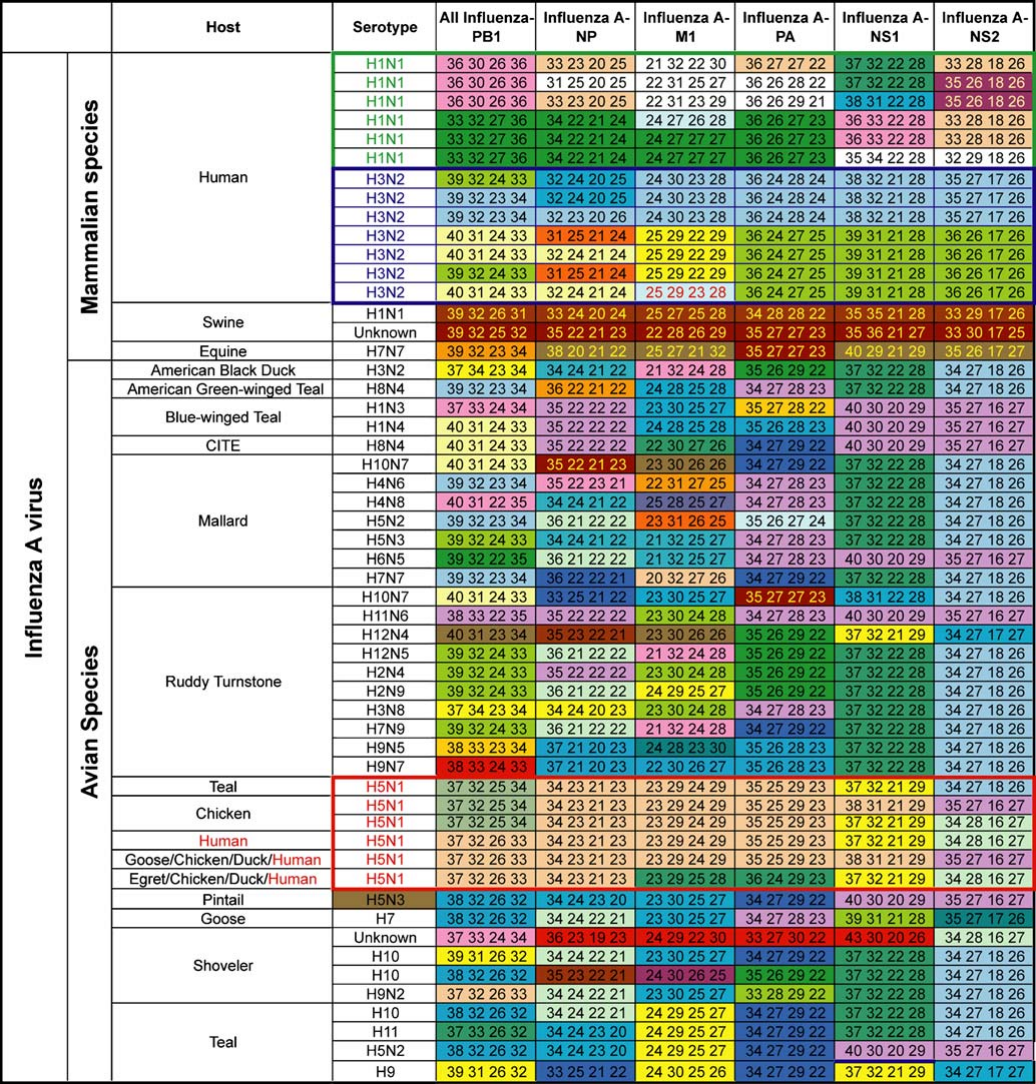
Detection and characterization of important human and avian influenza virus subtypes. Base composition signatures are shown in A, G, C, T order. Identical base compositions within a column are the same color. Base compositions represented only once are shown in white. Base compositions from human H1N1, human H3N2 and avian/human H5N1 isolates (in green, blue and red boxes, respectively) are included in Figure 2.

To measure the breadth of coverage and resolution offered by the panel of primers described in Methods (details in Table S1), we tested 92 well-characterized influenza virus isolates collected from human, avian, and animal species. Despite the extensive genetic diversity of this sample set, the broad-range primers generated amplicons from all isolates, and base composition signatures distinguished the isolates (Figure 1). Most isolates showed base compositions consistent with expected signatures for the corresponding H/N sub-types based on bioinformatic analysis of existing sequence data. Two of the isolates, however, showed previously unknown base compositions at several primer loci suggesting these might be novel influenza virus types; these are noted as “Unknown” in Figure 1.

**Figure 2:**
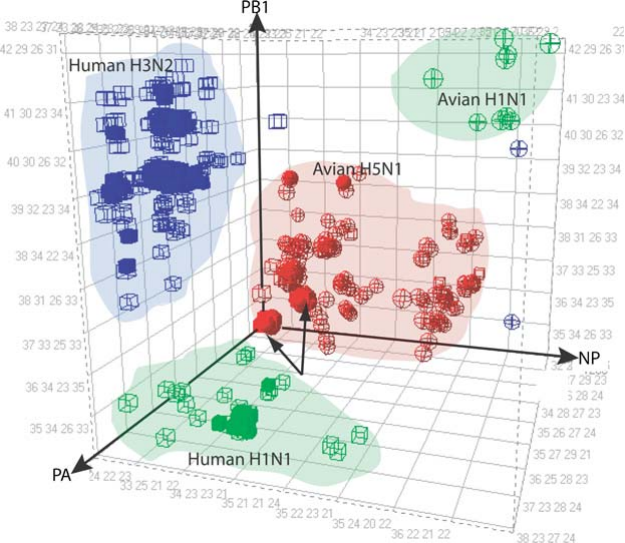
Spatial clustering of influenza virus subtypes. Each axis represents base composition bins (A, G, C, T) from a single primer pair. Solid symbols represent experimental measurements from this study, while open symbols are calculated base compositions determined from published sequences. Human isolates are shown as cubes and avian isolates as spheres. H1N1 isolates are shown in green, H3N2 in blue, and H5N1 in red. Arrows indicate avian influenza viruses isolated from humans.

Base composition signatures provide a multidimensional fingerprint of the genomes of the various viruses, which can be used to determine clusters of related species/sub-types. One such representation (Figure 2) shows base composition data derived from the PA, PB1, and NP gene segments analyzed on individual axes. Importantly, only three of the six influenza A primer pairs from Figure 1 are visualized on this three-dimensional plot. There was strong agreement between the bioinformatic analysis of sequence data from GenBank and experimental measurements of base composition signatures from Figure 1. Human H3N2 and H1N1 viruses clustered independently from each other and from the avian/human H5N1 and H1N1 viruses.

### RT-PCR/ESI-MS detection of influenza virus in human clinical specimens

To assess the utility of the RT-PCR/ESI-MS assay for surveillance of influenza virus in human populations, we analyzed 656 blinded clinical samples collected over a seven-year period (1999–2006). The results were compared with conventional analysis of the same samples by virus culture/serology and real-time RT-PCR methods. Two hundred forty-three samples were influenza positive both by RT-PCR/ESI-MS and conventional assays. Ten samples were positive only by RT-PCR/ESI-MS while eight samples were positive only by culture/real-time RT-PCR, corresponding to approximately 97% sensitivity and 98% specificity. Of the influenza-positive samples, RT-PCR/ESI-MS analysis identified 186 as influenza A virus and 67 as influenza B virus, in complete agreement with conventional typing methods.

Base composition analysis of multiple RNA segments enables further categorization of isolates into previously established clades determined by sequencing (details shown in Table S2). Of the 186 influenza A samples, we determined that 149 of were H3N2 subtype and 34 were H1N1. The subtype of three samples could not be distinguished between H3N2 and H1N2 because these viruses probably arose from a recent reassortment of the H gene from an H1N1 virus with gene segments from an H3N2 virus[12]. Importantly, our predictions agreed completely with serology and direct RT-PCR analysis of the H and N segments from these samples. Nonetheless, although base composition analysis of the PB1, NP, M1, PA, and NS gene segments can be highly predictive of the H and N types, direct analysis of the H and N segments is necessary for unambiguous subtyping due to the potential for viral reassortment.

In addition to identification and species typing, RT-PCR/ESI-MS provided a quantitative estimate of the number of viral genome copies in the original patient sample. This was achieved by including a fixed amount (300 copies/well) of an internal RNA calibration standard in each PCR reaction[19]–[22]. The genome copy numbers in the influenza samples ranged from low (<100 genome copies per well) to intermediate (100–2,500 copies/well) to high (>3,000 copies per well). Based on the samples within the linear range of the calibration standard, we estimated an average genome load of 750 copies/PCR reaction, representing an average viral load of ∼1.5×10^4^ genomes from material extracted from the original swab or 200 µL of a nasal aspirate. Six of the samples required serial dilutions to obtain viral concentrations in the quantifiable spectrum and ranged from 1.5×10^6^ to 8.0×10^7^ genomes/swab. Thus, for influenza-positive patients with respiratory symptoms, we observed a range of five orders of magnitude in viral RNA shedding.

### RT-PCR/ESI-MS tracking of influenza virus evolution

**Figure 3:**
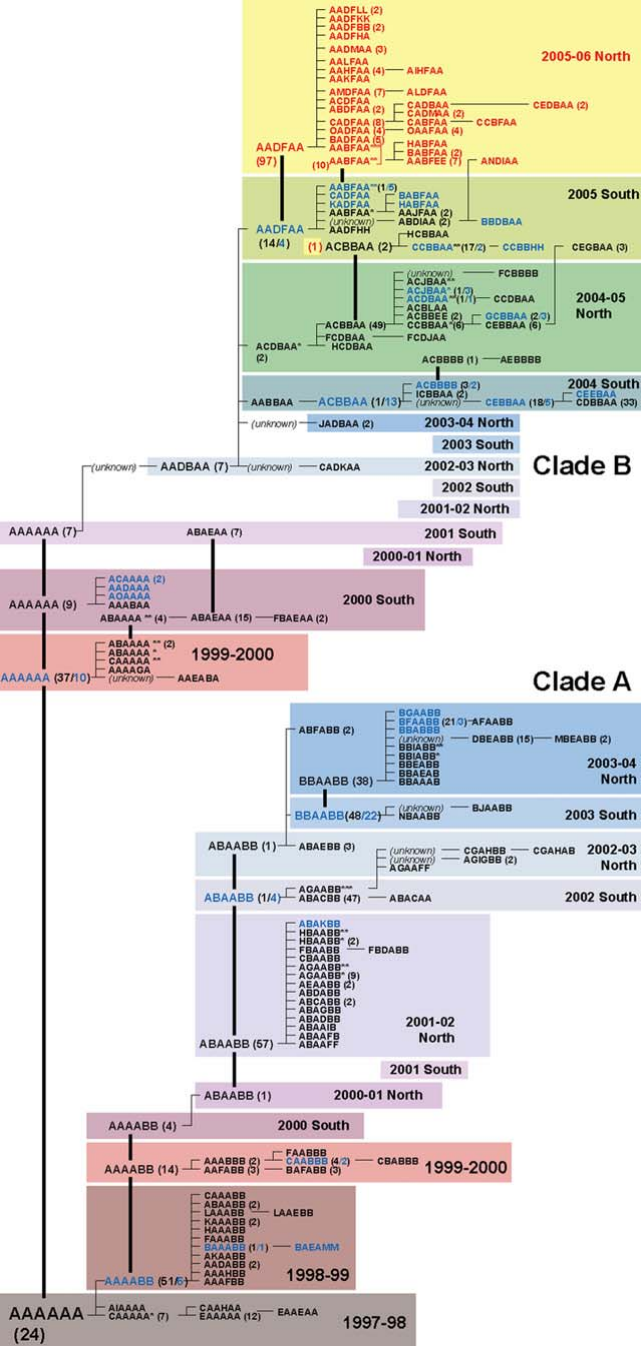
Clade distribution of H3N2 influenza viruses. Unique base composition types are reported using a six-letter code (see text) and are chronologically sorted bottom to top (color boxes, seasons 1997 to 2006). From year 2000 onwards, seasons were labeled “North” and “South” to reflect the northern or southern hemispheric origin of the samples. Thick vertical bars represent the persistence of main types between consecutive seasons. Within each season, the number of isolates is reported between parentheses for types encountered more than once. Thin horizontal lines represent the spawning of new types through the accumulation of single mutations (left to right). Black font: types determined through sequence analysis; blue font: experimentally determined base composition types; red font: experimentally determined base composition types for season 2005–06. Ten rare sequence types (∼1.5%) were not uniquely discernable by the base composition analysis of the eight amplicons used in this analysis, as more than one subtype produced the same BC-type. These BC-types are indicated by asterisks.

To demonstrate the capabilities of RT-PCR/ESI-MS to track the evolution of circulating influenza viruses, we created a tree representation of the H3N2 influenza virus sequences from Genbank (Figure 3, black) as described in Methods. The 104 experimentally determined H3N2 base compositions were mapped onto this tree (Figure 3, blue). Analysis revealed a distribution very similar to the sequence-derived clades of Holmes et al.[12]. The branch terminating in clade A represents the dominant branch between years 1999–2004, with a clade B branch that co-circulates in the same time period. Strikingly, however, clade-A isolates were not detected after this time and only clade-B viruses were found circulating world-wide post the 2003–2004 season, as determined by both viral sequence entries in GenBank and our experimental analysis of patient samples.

**Figure 4:**
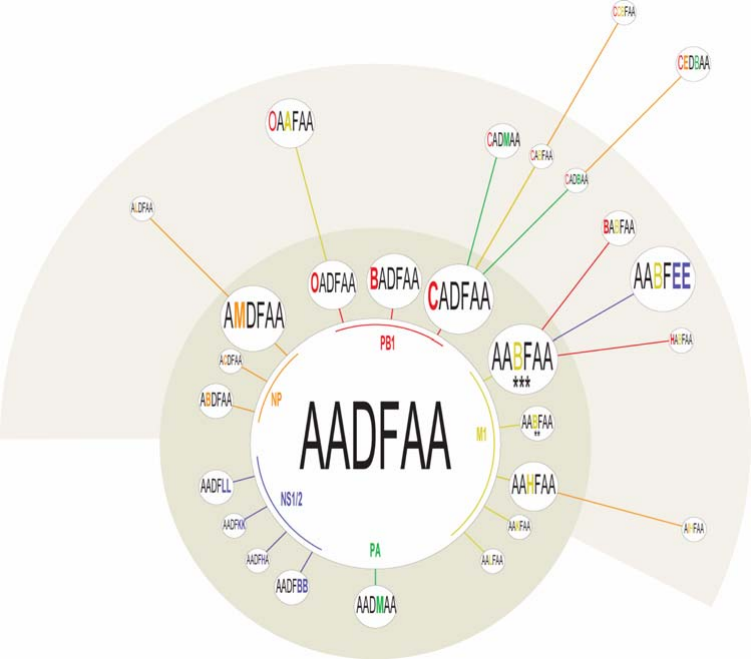
Relationship of founder isolate AADFAA and closest descendents in the 2005–2006 season. The areas of the circles are scaled to the number of human samples that contained the BC-types. Each concentric ring represents a single, double and triple mutations removed from the founder isolate, color coded for the gene containing the mutation. The order of the letters in the BC-type correspond to the six primer pairs used in this study, targeting PB1, NP, PA, M1, NS1 and NS2, respectively.

The tree was further expanded by the addition of 174 influenza A H3N2 base compositions, resulting in 29 distinct base composition types (BC types), from samples collected in North America during the 2005–2006 season (Figure 3, red). The most abundant BC type observed in 2005–2006 (AADFAA, 97 instances) was also one of the more abundant BC types from the previous winter flu season in the southern hemisphere (e.g., A/Canterbury/125/2005; Figure 3, 2005 South), suggesting that this BC type from the southern hemisphere might have been the founder of the 2005–2006 BC types in the northern hemisphere. Most of the other BC types observed in the 2005–2006 season were within one or two mutations of this major BC type, suggesting that these might have been derived from AADFAA through mutations during the 2005–2006 season. A second BC-type (AABFAA), which differs from AADFAA by a single mutation in the M1 locus, was also observed in the 2005 South and 2005–2006 North seasons and was potentially the founder of several additional BC types observed in 2005–2006. The locations of the mutations within each target locus responsible for base composition changes observed in the 2005–2006 northern isolates were verified by direct sequencing of the PCR products (results shown in Table S3) and confirmed the findings described above. A visual display of the most likely relationships among the isolates is shown in Figure 4. Collectively, these results demonstrate the richness of the genetic information provided by direct RT-PCR/ESI-MS analysis of human clinical materials.

### Detection of infections with more than one virus and emerging genetic variants

**Figure 5:**
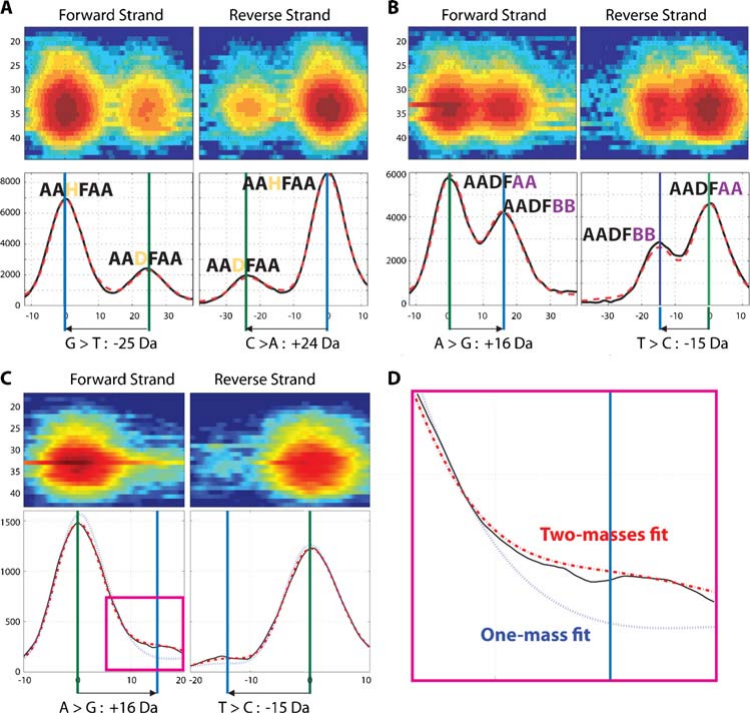
Detection of mixed viral populations. Panels A, B, and C are representations of mass spectral data. The heat maps in the top sections are a charge state representation of the data; the spectral plots in the lower sections were created by filtering the charge state responses to create signal representations vs. mass. The main peaks on the spectral plots are the primary amplicons and appear as hot spots in the charge state representations; the secondary amplicons appear as “cloudy” regions to the right and left for the forward and reverse strands, respectively. Panels A and B contain two species in relatively large ratios (20–50% mixtures) and involve the season 2005–2006 parent BC-type (AADFAA) and a type with a single mutation (panel A, within the M1 amplicon, BC-type AAHFAA; panel B, within the overlapping NS1 and NS2 amplicons, BC-type AADFBB). Panel C shows detection of a low abundance type (2–5%). Panel D shows a close-up view of the mass spectrum from Panel C. In this view, the shoulder of the peak is fit with a single mass model (blue dotted line) and a two mass model (dashed red line).

Infections with more than one influenza virus type can be identified with high sensitivity by RT-PCR/ESI-MS. Figures 5A and 5B show spectra from two clinical samples, each containing a mixture of two different viral BC types observed in samples collected during the 2005–2006 season. Figures 5A and B show the spectra from samples containing a mixture of the “parent” BC-type AADFAA and single-nucleotide variations AAHFAA and AADFBB, respectively, providing a snapshot of enduring “fit” quasispecies contributors and of potential viral evolution in action.

The dynamic range for mixed RT-PCR/ESI-MS detections has previously been determined to be approximately 100∶1[20], which allows for detection of viral variants with as low as 1% abundance in a mixed population. Figures 5C and 5D show results obtained with a clinical sample containing a mixture of viruses at the limit of detection for mixed populations. To demonstrate that these peaks truly represented mixed viral populations, the PCR amplicons were cloned and 450 independent colonies were sequenced. Nine (2%) of these 450 clones had the predicted mutations, correlating well with the measured amplitude of the low-abundance peaks.

A total of 293 non-overlapping nucleotides, excluding the primer regions, were analyzed using the genetic loci targeted by the influenza A primers. This corresponds to 2.15% of the influenza A virus genome. Out of 174 human samples analyzed from the 2005–2006 season, only two showed evidence for mixed viral populations at one of the six loci, corresponding to 1.1% of the samples. Thus, assuming the same mutation rate for the broader viral genome as for the region analyzed by PCR/ESI-MS, about 50% of the human H3N2 virus samples would have a mixed population of viruses.

## Discussion

Choosing amongst the various molecular methods available for pandemic influenza surveillance requires consideration of both practical issues (e.g., broad availability, convenience, cost, and throughput) and scientific issues relevant to public health (e.g., sensitivity, breath of coverage, and the depth and value of the information provided). At one end of the spectrum, a conventional RT-PCR test with specific primers and probes provides a highly-specific, sensitive, rapid, convenient, quantitative, relatively inexpensive, and high-throughput format that can provide valuable surveillance information. However, these tests are not optimal for surveillance when the exact nature of the pandemic virus is not known. Moreover, without supplemental nucleic acid sequencing, conventional RT-PCR-based tests are not capable of signaling the appearance of new genetic variants, except by potentially demonstrating a loss of sensitivity. Further, a single RT-PCR test can achieve only a single presence/absence analysis limited to the specific target for which it was designed. Discrimination of all known variants of influenza at the level of resolution described here would require hundreds of independent RT-PCR reactions. At the other end of the spectrum, virus isolation using culture methods followed by complete genome sequencing does not require prior knowledge of the virus' sequence and provides clade-level resolution and highly detailed information regarding virus evolution. Unfortunately, this method is slow, labor intensive, expensive, and low throughput, rendering it ineffective in public health arenas requiring rapid response.

In this work we describe a novel method that employs some of the best properties of each of the discussed techniques, and also supplies additional valuable information not provided by those techniques. For example, our method may identify mixed populations of viruses, either as viral quasispecies as previously illustrated (i.e., development of “drift” strains) or co-infections with circulating strains (i.e., potential for development of “shift” strains). Recent advances in ESI-MS using bench-top mass spectrometers have enabled analysis of PCR amplicons with sufficient mass accuracy that the nucleotide base composition (the A, G, C, and T count) of the PCR amplicon can be unambiguously determined[23]. The approach outlined here provides two important advantages for surveillance. First, broad-range primers targeted to highly-conserved sites within the influenza virus family that flank highly variable, information-rich regions can be used to amplify sequences from highly diverse viruses in the same assay. The measured base compositions allow identification of the viral species with a high degree of resolution. For this approach, we selected primer pairs targeting the core gene segments (PB1, PB2, PA, M, NS, and NP) that have conserved regions spanning all known influenza viruses and used the resultant base compositions to infer the influenza virus H/N types. In silico analysis of influenza virus sequences from the GenBank database showed that this approach would detect all known influenza viruses and distinguish >90% of all species and types. This overcomes the limitations of directly targeting the H and N segments, which requires specific primer pairs for each known H/N type. Further, since the H/N segments evolve rapidly, newly emerging strains might not be readily detected by the traditional approach.

Second, mixed viral populations in the same sample that differ only by a single mutation and is present in as low as 1–2% of the virus population can be identified, providing early insights into viral evolution as an integral component of surveillance. This information-rich result is provided with the same throughput and consumable costs as conventional, sequence-specific RT-PCR assays.

The results from 174 influenza A H3N2 samples from the 2005–2006 season in the northern hemisphere were particularly interesting because they revealed viral evolution during a single season. The viruses detected appeared to have been seeded from two of the more abundant BC types circulating during the previous season in the southern hemisphere. The majority (97) of the samples had identical BC types and probably arose from a single founder from the previous season in the southern hemisphere. Most of the remaining samples differed from this founder BC type by one or two additional point mutations within the target regions described here. Surprisingly, when mutations occurred, they became fixed rapidly in the viral population, since only two of the 174 samples from the 2005–2006 season showed evidence of mixed populations. Sequencing revealed that both mutations were silent transitions in third codon positions.

In summary, the RT-PCR/ESI-MS method has the capacity to provide rapid diagnosis of human influenza in individual patients with respiratory symptoms, as well as public health surveillance of emerging, potentially pandemic strains, including novel reassortants. The use of RT-PCR/ESI-MS for human as well as avian/animal surveillance offers the potential for new insights into viral evolution on a scale and at a cost previously not possible. Bench-top mass spectrometers are capable of analyzing complex PCR products at a rate of approximately one reaction product/minute, making the RT-PCR/ESI-MS technology practical for large-scale analysis of clinical specimens or for animal surveillance. Further, as we have demonstrated with influenza viruses, this method provides sensitive detection directly from patient specimens with specificity approximating sequence-level resolution. The ability to quantitate virus shedding, detect low-abundance, mixed infections, and identify new genetic variants without prior knowledge of viral sequence also make this technology ideally suited for monitoring the emergence of drug-resistance mutations during therapy or for identifying newly emerging antigenic variants.

## Materials and Methods

### Viral samples

Eighteen human influenza virus A (H1N1, H3N2) and six human influenza virus B isolates were obtained as tissue culture fluids from the Naval Health Research Center (NHRC, San Diego, CA). NHRC also supplied 336 human respiratory specimens (240 throat swabs, 26 nasal swabs, and 70 nasal wash specimens) collected and archived from various U.S. military bases around the country from 1999 through 2005. The New York State Department of Health, Wadsworth Center (Albany, NY) supplied 100 respiratory specimens collected between 1999–2005 (88 nasopharyngeal aspirates; 12 assorted nasal aspirates, BAL, tracheal aspirates, and throat swabs). Johns Hopkins University (JHU, Baltimore, MD) provided 229 nasal aspirates collected during 2003–2005. The Texas Department of State Health Services provided a total of 574 assorted throat, sputum, and nasopharyngeal aspirate specimens collected from 2005 to 2006. The 63 avian isolates from 16 different avian species were obtained from the United States (The University of Georgia collection), Egypt, and Asia/Middle East (Naval Medical Research Unit #3 (NAMRU-3), Cairo, Egypt). NAMRU-3 also provided all of the HPAI (avian H5N1) isolates collected from 2006 outbreaks in Egypt, Iraq, and Central Asia. Equine (VR-297) and swine isolates (VR-333) were obtained from the American Type Culture Collection (ATCC). Additional swine isolates were provided by Dr. Gregory Gray from the University of Iowa. All samples were collected under appropriate human or animal use protocols, or from granted exempt use of anonymous clinical specimens, from the respective organizations.

### Sample processing

Viral stock samples consisting of cultured virus or stocks obtained from ATCC were prepared for analysis using the Qiagen QiaAmp Virus kit (Valencia, CA). Both manual (mini spin) kits and BioRobot kits were used. Robotic-based isolations were done on both the Qiagen MDx and Qiagen BioRobot 8000 platforms. Clinical swab samples were stored in Viral Transport Media (VTM). VTM solution (1 ml) was passed over a 0.2 micron filter, which was then subjected to bead beating in a small amount of lysis buffer. The resulting viral lysate was then used following the same protocol as above.

### Primer design

Based upon analysis of multiple influenza sequence alignments, pan-influenza virus RT-PCR primer sets were developed that were capable of amplifying all three influenza virus species (A, B, and C) and subtypes (HxNy) from different animal hosts (e.g., human, avian, and swine) and distinguishing essential molecular features using base composition signatures. Additional primer pairs were designed that broadly amplified all known members of a particular species, but that did not cross-amplify members of different species (e.g., pan-influenza A and pan-influenza B primers). A surveillance panel of eight primer pairs (Table S1) was selected comprising one pan-influenza primer pair targeting the PB1 segment, five pan-influenza A primer pairs targeting NP, M1, PA, and the NS segments, and two pan-influenza B primer pairs targeting NP and PB2 segments. All primers used in this study had a thymine nucleotide at the 5′ end to minimize addition of non-templated adenosines during amplification using Taq polymerase[24]. The sensitivity of each RT-PCR primer pair was determined using known quantities of a synthetic calibrant RNA template as described previously[20]. Each of the primer pairs was sensitive to as few as twenty copies of the calibrant RNA and several primers were sensitive to five copies (Table S1).

### Reverse transcription PCR (RT-PCR)

One-step RT-PCR was performed in a reaction mix consisting of 4 U of Ampli*Taq* Gold (Applied Biosystems, Foster City, CA); 20 mM Tris, pH 8.3; 75 mM KCl; 1.5 mM MgCl_2_; 0.4 M betaine; 800 µM mix of dATP, dGTP, dCTP, and dTTP (Bioline USA Inc., Randolph, MA); 10 mM dithiothreitol; 100 ng sonicated polyA DNA (Sigma Corp., St Louis, MO); 40 ng random hexamers (Invitrogen Corp.); 1.2 U Superasin (Ambion Corp, Austin, TX); 400 ng T4 gene 32 protein (Roche Diagnostics Corp., Indianapolis, IN); 2 U Superscript III (Invitrogen Corp, Carlsbad CA.); 20 mM sorbitol (Sigma Corp.); and 250 nM of each primer. The following RT-PCR cycling conditions were used: 60°C for 5 min, 4°C for 10 min, 55°C for 45 min, 95°C for 10 min, followed by 8 cycles of 95°C for 30 seconds, 48°C for 30 seconds, and 72°C for 30 seconds, with the 48°C annealing temperature increasing 0.9°C each cycle. The PCR was then continued for 37 additional cycles of 95°C for 15 seconds, 56°C for 20 seconds, and 72°C for 20 seconds. The RT-PCR cycle ended with a final extension of 2 min at 72°C followed by a 4°C hold.

### Mass spectrometry and base composition analysis

The RT-PCR products were analyzed using the Ibis T5000 universal biosensor platform (Ibis Biosciences, Inc., Carlsbad, CA; http://www.ibisbiosciences.com), which performs automated post-PCR desalting, ESI-MS signal acquisition, spectral analysis, and data reporting as described previously[23]. Briefly, the steps were as follows: 15 µL aliquots of each PCR reaction were desalted and purified using a weak anion exchange protocol as described elsewhere[25]. Accurate mass (±1 ppm), high-resolution (M/dM>100,000 FWHM) mass spectra were acquired for each sample using high-throughput ESI-MS protocols described previously[20]. For each sample, approximately 1.5 µL of analyte solution was consumed during the 74-second spectral acquisition. Raw mass spectra were post-calibrated with an internal mass standard and deconvolved to monoisotopic molecular masses. Unambiguous base compositions were derived from the exact mass measurements of the complementary single-stranded oligonucleotides[26]. Quantitative results are obtained by comparing the peak heights with an internal PCR calibration standard present in every PCR well at 100 molecules[20].

### Phylogenetic analysis

To demonstrate the capabilities of RT-PCR/ESI-MS to track the evolution of circulating influenza viruses, we used a bioinformatic approach to develop a framework on which to display the RT-PCR/ESI-MS results obtained with H3N2 viruses. Complete genome sequences of all H3N2 human influenza viruses available in GenBank were analyzed. A total of 731 genomes were included, from which we inferred the phylogeny of H3N2 influenza virus since 1996. Using the 565-nucleotide concatenated sequence of the six loci that we analyzed by RT-PCR/ESI-MS, we constructed a non-redundant alignment of 105 sequence types. Base compositions were determined for each genome segment (i.e., locus) analyzed and each unique base composition at each of these loci was assigned a letter according to decreasing number of occurrences (therefore, the letter A represents the most common allele identified at each locus). The concatenation of the six base-composition letters from the PB1, NP, M1, PA, NS1, and NS2 loci from the sequences of 731 H3N2 viruses (1996–2005) available in GenBank yielded 95 six-letter codes referred to as base composition types (BC types). The predominant type is labeled AAAAAA. The topology of the tree was then deduced from the alignment of non-redundant sequences using the programs *dnadist* and *neighbor* from the Phylip package (http://evolution.genetics.washington.edu/phylip.html). Since the sequence types differ mostly by discrete single mutations, the original computer-generated tree was then extensively edited using graphics tools to place the labels of intermediate types within the tree itself in lieu of zero-length branches. Readability was further improved by sorting parallel branches chronologically.

## Supporting Information

Table S1 Influenza virus primer pairs used in this study. Genbank reference sequence for each segment is indicated; however, the primer sequences are not identical to the reference sequence as described in the Methods. The limits of detection for each RT-PCR primer pair were determined using known quantities of a synthetic calibrant RNA template.
(0.06 MB PDF)

Table S2 Distribution of BC-types observed in influenza A H3N2 positive human respiratory samples. Unique base compositions at each genome segment locus analyzed were assigned letter codes and concatenation of letter codes across the six loci analyzed yielded BC-types. H1N1 samples were not assigned a BC-type. Experimentally determined BC-types (marked RT-PCR/ESI-MS Analysis results) were compared to BC-type signature information of sequences currently available in GenBank and the closest matching strain is shown (right pane). Last column shows comparison to clade designation described in Holmes et al [12].
(0.24 MB PDF)

Table S3 Mutations observed in influenza virus isolates in 2005–06 season. Out of the 174 influenza A H3N2 positive samples analyzed from the 2005–2006 season, 29 genotypes were assigned based on RT-PCR/ESI-MS and 30 were assigned based on sequencing. There were no instances of “ESI-MS silent” compensating double mutations (e.g., simultaneous A>G and G>A within the same amplicon leading to no change in the observed BC-type) as verified by sequencing.
(0.10 MB PDF)
