# Supplementary material for: Global Surveillance of Emerging Influenza Virus Genotypes by Mass Spectrometry
Source: PLoS One. 2007 May 30;2(5):e489. doi: 10.1371/journal.pone.0000489 (PMC1876795; doi:10.1371/journal.pone.0000489)
Supplement: Table S1 — Influenza virus primer pairs used in this study. Genbank reference sequence for each segment is indicated; however, the primer sequences are not identical to the reference sequence as described in the Methods. The limits of detection for each RT-PCR primer pair were determined using known quantities of a synthetic calibrant RNA template. (0.06 MB PDF) [file pone.0000489.s001.pdf]

**Table S1: Influenza virus primer pairs used in this study**. Genbank reference sequence for each segment is indicated; however, the primer sequences are not identical to the reference sequence as described in the Methods. The limits of detection for each RT-PCR primer pair were determined using known quantities of a synthetic calibrant RNA template.

| Primer Number | Target Segment | Target Species | Genbank Reference Sequence | | Primer Sequence (5'→3') | LOD (copy numbers) |
|---|---|---|---|---|---|---|
| 2798 | PB1 | Pan Influenza | J02151 | F<br>R | TGTCCTGGAATGATGATGGGCATGTT<br>TCATCAGAGGATTGGAGTCCATCCC | 5 |
| 1266 | Nuc | Influenza A | J02147 | F<br>R | TACATCCAGATGTGCACTGAACTCAAACTCA<br>TCGTCAAATGCAGAGAGCACCATTCTCTCTA | 20 |
| 1279 | M1 | Influenza A | NC_004524 | F<br>R | TCTTGCCAGTTGTATGGGCCTCATATAC<br>TGGGAGTCAGCAATCTGCTCACA | 20 |
| 1287 | PA | Influenza A | NC_004520 | F<br>R | TGGGATTCCTTTCGTCAGTCCGA<br>TGGAGAAGTTCGGTGGGAGACTTTGGT | 5 |
| 2775 | NS1 | Influenza A | NC_004525 | F<br>R | TCCAGGACATACTGATGAGGATGTCAAAAATGCA<br>TGCTTCCCCAAGCGAATCTCTGTA | 20 |
| 2777 | NS2 | Influenza A | NC_004525 | F<br>R | TGTCAAAAATGCAATTGGGGTCCTCATC<br>TCATTACTGCTTCTCCAAGCGAATCTCTGTA | 20 |
| 1261 | PB2 | Influenza B | NC_002205 | F<br>R | TCCCATTGTACTGGCATACATGCTTGA<br>TATGAACTCAGCTGATGTTGCTCCTGC | 5 |
| 1275 | Nuc | Influenza B | NC_002208 | F<br>R | TCCAATCATCAGACCAGCAACCCTTGC<br>TCCGATATCAGCTTCACTGCTTGTGG | 5 |
