# Supplementary material for: Global Surveillance of Emerging Influenza Virus Genotypes by Mass Spectrometry
Source: PLoS One. 2007 May 30;2(5):e489. doi: 10.1371/journal.pone.0000489 (PMC1876795; doi:10.1371/journal.pone.0000489)
Supplement: Table S2 — Distribution of BC-types observed in influenza A H3N2 positive human respiratory samples. Unique base compositions at each genome segment locus analyzed were assigned letter codes and concatenation of letter codes across the six loci analyzed yielded BC-types. H1N1 samples were not assigned a BC-type. Experimentally determined BC-types (marked RT-PCR/ESI-MS Analysis results) were compared to BC-type signature information of sequences currently available in GenBank and the closest matching strain is shown (right pane). Last column shows comparison to clade designation described in Holmes et al [12]. (0.24 MB PDF) [file pone.0000489.s002.pdf]

Table S2. Distribution of BC-types observed in influenza A H3N2 positive human respiratory samples. Unique base compositions at each genome segment locus analyzed were assigned letter codes and concatenation of letter codes across the six loci analyzed yielded BC-types. H1N1 samples were not assigned a BC-type. Experimentally determined BC-types (marked RT-PCR/ESI-MS Analysis results) were compared to BC-type signature information of sequences currently available in GenBank and the closest matching strain is shown (right pane). Last column shows comparison to clade designation described in Holmes et al(12).

Sample Data			PCR/ESI-MS Analysis results							Comparison to existing data		
Type	# of instances	Specimen Year	BC-type	PB1	NP	M1	PA	NS1	NS2	Closest Database Match	Season in circulation	Designation Holmes et al
H3N2	1	2005	BBDBAA	39 32 23 34	32 24 20 25	24 30 22 29	36 24 27 25	39 31 21 28	36 26 17 26	A/Western Australia/76/2005	2005	Clade B Variants
	1	2005	HABFAA	38 33 24 33	31 25 20 25	25 29 22 29	37 24 27 24	39 31 21 28	36 26 17 26	A/CANTERBURY/127/2005(H3N2)	2005	
	1	2006	BABFAA	39 32 23 34	31 25 20 25	25 29 22 29	37 24 27 24	39 31 21 28	36 26 17 26	A/CANTERBURY/127/2005(H3N2)	2005	
	5	2005	AABFAA	39 32 24 33	31 25 20 25	25 29 22 29	37 24 27 24	39 31 21 28	36 26 17 26	A/CANTERBURY/127/2005(H3N2)	2005	
	1	2006	CADFAA	40 31 24 33	31 25 20 25	24 30 22 29	37 24 27 24	39 31 21 28	36 26 17 26	A/CANTERBURY/129/2005(H3N2)	2005	
	1	2005	KADFAA	38 32 24 34	31 25 20 25	24 30 22 29	37 24 27 24	39 31 21 28	36 26 17 26	A/CANTERBURY/220/2005	2005	
	4	2005	AADFAA	39 32 24 33	31 25 20 25	24 30 22 29	37 24 27 24	39 31 21 28	36 26 17 26	A/CANTERBURY/129/2005(H3N2)	2005	
	1	2005	CCBBHH	40 31 24 33	31 25 21 24	25 29 22 29	36 24 27 25	40 30 21 28	37 25 17 26	A/CANTERBURY/186/2005(H3N2)	2005	
	2	2005	CCBBAA	40 31 24 33	31 25 21 24	25 29 22 29	36 24 27 25	39 31 21 28	36 26 17 26	A/CANTERBURY/186/2005(H3N2)	2005	
	3	2004	ACJBAA	39 32 24 33	31 25 21 24	26 28 22 29	36 24 27 25	39 31 21 28	36 26 17 26	A/NEW YORK/191/2005(H3N2)	2004-05	
	1	2005	ACDBAA	39 32 24 33	31 25 21 24	24 30 22 29	36 24 27 25	39 31 21 28	36 26 17 26	A/NEW YORK/243/2005(H3N2)	2004-05	
	3	2005	GCBBAA	41 30 24 33	31 25 21 24	25 29 22 29	36 24 27 25	39 31 21 28	36 26 17 26	A/NEW YORK/334/2004(H3N2)	2004-05	
	1	2005	CEEBAA	40 31 24 33	32 24 21 24	25 29 23 28	36 24 27 25	39 31 21 28	36 26 17 26	A/BAY OF PLENTY/279/2004(H3N2)	2004	
	5	2005	CEBBAA	40 31 24 33	32 24 21 24	25 29 22 29	36 24 27 25	39 31 21 28	36 26 17 26	A/BAY OF PLENTY/279/2004(H3N2)	2004	
	2	2004	ACBBBB	39 32 24 33	31 25 21 24	25 29 22 29	36 24 27 25	38 32 21 28	35 27 17 26	A/ASHBURTON/280/2004(H3N2)	2004	
	13	2004	ACBBAA	39 32 24 33	31 25 21 24	25 29 22 29	36 24 27 25	39 31 21 28	36 26 17 26	A/CANTERBURY/104/2005(H3N2)	2004	
	2	1999	ACAAAA	39 32 24 33	31 25 21 24	24 30 23 28	36 24 28 24	39 31 21 28	36 26 17 26	A/CANTERBURY/17/2000(H3N2)	2000	
	1	1999	AADAAA	39 32 24 33	31 25 20 25	24 30 22 29	36 24 28 24	39 31 21 28	36 26 17 26	A/NEW YORK/139/1999(H3N2)	1999-2000	
	1	2000	AOAAAA	39 32 24 33	31 26 20 24	24 30 23 28	36 24 28 24	39 31 21 28	36 26 17 26	A/NEW YORK/139/1999(H3N2)	1999-2000	
	10	1999	AAAAAA	39 32 24 33	31 25 20 25	24 30 23 28	36 24 28 24	39 31 21 28	36 26 17 26	A/NEW YORK/139/1999(H3N2)	1999-2000	1999-2000
H1N2	3	1999	AAAAAA	39 32 24 33	31 25 20 25	24 30 23 28	36 24 28 24	39 31 21 28	36 26 17 26	A/NEW YORK/139/1999(H3N2)	1999-2000	1999-2000
H3N2	1	2003	BGAABB	39 32 23 34	33 23 20 25	24 30 23 28	36 24 28 24	38 32 21 28	35 27 17 26	A/NEW YORK/14/2003(H3N2)	2003-04	Clade A
	3	2004	BFAABB	39 32 23 34	32 23 20 26	24 30 23 28	36 24 28 24	38 32 21 28	35 27 17 26	A/NEW YORK/14/2003(H3N2)	2003-04	
	1	2002	BBABBB	39 32 23 34	32 24 20 25	24 30 23 28	36 24 27 25	38 32 21 28	35 27 17 26	A/NEW YORK/50/2003(H3N2)	2003-04	
	22	2004	BBAABB	39 32 23 34	32 24 20 25	24 30 23 28	36 24 28 24	38 32 21 28	35 27 17 26	A/CANTERBURY/382/2003(H3N2)	2003	
	4	2002	ABAABB	39 32 24 33	32 24 20 25	24 30 23 28	36 24 28 24	38 32 21 28	35 27 17 26	A/CANTERBURY/72/2002(H3N2)	2002	Seasons 2001-03
	1	unknown	ABAKBB	39 32 24 33	32 24 20 25	24 30 23 28	36 24 26 26	38 32 21 28	35 27 17 26	A/CANTERBURY/72/2002(H3N2)	2002	Seasons 2001-03
	2	1999	CAABBB	40 31 24 33	31 25 20 25	24 30 23 28	36 24 27 25	38 32 21 28	35 27 17 26	A/NEW YORK/137/1999(H3N2)	1999-2000	1999 Outliers
	1	2000	BAEAMM	39 32 23 34	31 25 20 25	25 29 23 28	36 24 28 24	39 32 20 28	36 27 16 26	A/NEW YORK/261/1999(H3N2)	1998-99	1999-2000
	1	1999	BAAABB	39 32 23 34	31 25 20 25	24 30 23 28	36 24 28 24	38 32 21 28	35 27 17 26	A/NEW YORK/261/1999(H3N2)	1998-99	1999-2000
	6	1999	AAAABB	39 32 24 33	31 25 20 25	24 30 23 28	36 24 28 24	38 32 21 28	35 27 17 26	A/NEW YORK/140/1999(H3N2)	1998-99	1999-2000
H1N1	13	2001	Not Applicable	33 32 27 36	34 22 21 24	24 27 26 28	36 26 27 23	36 33 22 28	33 28 18 26	A/NEW YORK/233/2000(H1N1)	2001	Not Applicable
	2	2001		33 32 27 36	34 22 21 24	24 27 26 28	36 26 28 22	36 33 22 28	33 28 18 26	A/NEW YORK/350/2003(H1N1)	2003	
	1	2001		33 32 27 36	34 22 21 24	24 27 27 27	35 27 27 23	36 33 22 28	33 28 18 26	A/NEW YORK/212/2001(H1N1)	2001	
	9	2001		33 32 27 36	34 22 21 24	24 27 27 27	36 26 27 23	36 33 22 28	33 28 18 26	A/NEW YORK/208/2001(H1N1)	2001	
	6	2003		33 32 27 36	34 22 21 24	24 27 27 27	36 26 28 22	36 33 22 28	33 28 18 26	A/NEW YORK/205/2001(H1N1)	2001	
	1	2001		33 32 27 36	34 22 21 24	24 27 27 27	36 26 27 23	36 34 22 27	33 29 18 25	A/NEW YORK/208/2001(H1N1)	2001	
	1	2001		32 33 27 36	34 22 21 24	24 27 27 27	36 26 27 23	36 33 22 28	33 28 18 26	A/NEW YORK/312/2001(H1N1)	2001	
	1	1999		33 32 27 36	35 22 21 23	24 28 27 26	36 26 28 22	37 32 21 29	32 29 18 26	A/NEW YORK/205/2001(H1N1)	2001	
