# Supplementary material for: Global Surveillance of Emerging Influenza Virus Genotypes by Mass Spectrometry
Source: PLoS One. 2007 May 30;2(5):e489. doi: 10.1371/journal.pone.0000489 (PMC1876795; doi:10.1371/journal.pone.0000489)
Supplement: Table S3 — Mutations observed in influenza virus isolates in 2005–06 season. Out of the 174 influenza A H3N2 positive samples analyzed from the 2005–2006 season, 29 genotypes were assigned based on RT-PCR/ESI-MS and 30 were assigned based on sequencing. There were no instances of “ESI-MS silent” compensating double mutations (e.g., simultaneous A>G and G>A within the same amplicon leading to no change in the observed BC-type) as verified by sequencing. (0.10 MB PDF) [file pone.0000489.s003.pdf]

Table S3. Mutations observed in influenza virus isolates in 2005-06 season.[1] Out of the 174 influenza A H3N2 positive samples analyzed from the 2005-2006 season, 29 genotypes were assigned based on RT-PCR/ESI-MS and 30 were assigned based on sequencing. There were no instances of "ESI-MS silent" compensating double mutations (e.g., simultaneous A>G and G>A within the same amplicon leading to no change in the observed BC-type) as verified by sequencing.

BC-type	# of isolates	# of mutations	PB1	NP	M1	PA	NS1	NS2
AADFAA	97	0	wt	wt	wt	wt	wt	wt
AABFAA	10	1	wt	wt	G→A_{34}	wt	wt	wt
			wt	wt	G→A_{70}	wt	wt	wt-(ns)
AAHFAA	4	1	wt	wt	G→T_{58}	wt	wt	wt
AAKFAA	1	1	wt-(ns)	wt	T→G_{43}	wt	wt	wt
AALFAA	1	1	wt-(ns)	wt-(ns)	A→G	wt-(ns)	wt-(ns)	wt-(ns)
BADFAA	5	1	C→T_{99}	wt	wt	wt	wt	wt-(ns)
CADFAA	8	1	G→A_{72}	wt	wt	wt	wt	wt
OADFAA	4	1	T→A_{69}	wt-(ns)	wt	wt	wt-(ns)	wt-(ns)
AMDFAA	7	1	wt	C→A_{46}	wt	wt	wt	wt
ACDFAA	1	1	wt	T→C_{49}	wt	wt	wt-(ns)	wt-(ns)
AADMAA	3	1	wt-(ns)	wt-(ns)	wt-(ns)	A→G	wt-(ns)	wt-(ns)
AADFBB	2	1	wt	wt	wt	wt	A→G	A→G_{31}
AADFLL	2	1	wt	wt-(ns)	wt	wt	C→T	C→T_{74}
AADFKK	1	1	wt-(ns)	wt-(ns)	wt-(ns)	wt	G→T	G→T
AADFHA	1	1	wt-(ns)	wt-(ns)	wt-(ns)	wt	G→A	wt-(ns)
ABDFAA	2	1	wt-(ns)	G->A_{45}	wt	wt	wt-(ns)	wt-(ns)
ALDFAA	1	2	wt	G→A_{48} + C→A_{46}	wt	wt	wt	wt-(ns)
HABFAA	1	2	A→G	wt-(ns)	G→A	wt	wt-(ns)	wt-(ns)
BABFAA	2	2	C→T (ns)	wt-(ns)	G→A_{34}	wt	wt	wt
CABFAA	1	2	G→A (ns)	wt-(ns)	G→A_{34}	wt	wt-(ns)	wt-(ns)
CADMAA	2	2	G→A_{57}	wt-(ns)	wt	A→G_{45}	wt-(ns)	wt
CADBAA	1	2	G→A_{57}	wt	wt	A→T_{61}	wt	wt
OAAFAA	4	2	T→A_{69}	wt	T→C_{52}	wt	wt	wt
AABFEE	7	2	wt	wt	G→A_{34}	wt	C→A_{95}	C→A_{74}
ANDIAA	1	2	wt-(ns)	G→T	wt-(ns)	G→A	wt-(ns)	wt-(ns)
AIHFAA	1	2	wt-(ns)	C→T	G→T	wt	wt-(ns)	wt-(ns)
CCBFAA	1	3	G→A_{72}	T→C	G→A_{34}	wt	wt	wt
ACBBAA	1	3	wt-(ns)	G→A	G→A	A→T	wt-(ns)	wt-(ns)
CEDBAA	2	4	G→A_{57}	G→A_{48} + T→C_{37}	wt	A→T_{61}	wt-(ns)	wt

[1] Abbreviations: wt, wild type (as observed in type AADFAA); ns, not sequenced.
